# First Confirmed Description of *Acremonium egyptiacum* from Greece and Molecular Identification of *Acremonium* and *Acremonium*-like Clinical Isolates

**DOI:** 10.3390/jof10090664

**Published:** 2024-09-21

**Authors:** Michael Arabatzis, Philoktitis Abel, Eleni Sotiriou, Aristea Velegraki

**Affiliations:** 1 First Department of Dermatology and Venereology, Medical School, Aristotle University, 546 43 Thessaloniki, Greece; epsotiri@auth.gr; 2 Phoenix Group, Birmingham B47 6WG, UK; therealphilabel@gmail.com; 3 Research Mycology Laboratory, Medical School, National and Kapodistrian University of Athens, 115 27 Athens, Greece; aveleg@med.uoa.gr; 4 Bioiatriki Healthcare Group, 115 26 Athens, Greece

**Keywords:** acremonium-like fungi, *Acremonium egyptiacum*, Greece, ITS, antifungal susceptibility, CLSI M38 Ed3

## Abstract

*Acremonium* and the recently separated acremonium-like genera, such as *Sarocladium*, are emerging causes of opportunistic disease in humans, mainly post-traumatic infections in immunocompetent hosts, but also invasive infections in immunocompromised patients, such as those undergoing transplantation. *Acremonium egyptiacum* has emerged as the major pathogenic *Acremonium* species in humans, implicated mainly in nail but also in disseminated and organ specific infections. In this first study of acremonium-like clinical isolates in Greece, 34 isolates were identified and typed by sequencing the internal transcribed spacer, and their antifungal susceptibility was determined by a modified CLSI standard M38 3rd Edition method for filamentous fungi. *A. egyptiacum* was the primary species (18 isolates) followed by *Sarocladium kiliense* (8), *Acremonium charticola*, *Gliomastix polychroma*, *Proxiovicillium blochii*, *Sarocladium terricola*, *Sarocladium zeae*, and *Stanjemonium dichromosporum* (all with one isolate). Two isolates, each with a novel ITS sequence, possibly represent undescribed species with an affinity to *Emericellopsis*. All three *A. egyptiacum* ITS barcode types described to date were identified, with 3 being the major type. Flutrimazole, lanoconazole, and luliconazole presented the lower minimum inhibitory concentration (MIC) values against *A. egyptiacum*, with a geometric mean (GM) MIC of 2.50, 1.92, and 1.57 μg/mL, respectively. Amphotericin B, itraconazole, posaconazole, voriconazole, terbinafine, amorolfine, and griseofulvin MICs were overall high (GM 12.79–29.49 μg/mL). An analysis of variance performed on absolute values showed that flutrimazole, lanoconazole, and luliconazole were equivalent and notably lower than those of all the other drugs tested against *A. egyptiacum*. Antifungal susceptibility of the three different *A. egyptiacum* genotypes was homogeneous. Overall, the high MICs recorded for all systemically administered drugs, and for some topical antifungals against the tested *A. egyptiacum* and other acremonium-like clinical isolates, justify the routine susceptibility testing of clinical isolates.

## 1. Introduction

The anamorphic genus *Acremonium* and the recently separated acremonium-like genera, such as *Sarocladium*, comprise fungal species that are primarily living in the environment, either saprobically in soil and decaying plant material or endophytically in plants [1,2,3,4]. Some of these species are capable of causing opportunistic disease in humans, mainly post-traumatic infections in immunocompetent hosts, but also emerging invasive infections in immunocompromised patients, such as those undergoing transplantation [2,5,6]. *Acremonium* species are thus established causes of post-traumatic infections such as keratitis and mycetoma, and are increasingly isolated from blood, central nervous system, sinus, bone/joint, pleura, peritoneum, and disseminated infections [3,5,6]. Additionally, they constitute an important cause of non-dermatophytic nail infections (onychomycosis) [7]. *Acremonium* has been found highly polyphyletic [8,9], and its current taxonomy is very complex, with acremonium-like fungi assigned to 63 genera, and 14 families [9]. It is practically impossible to identify the various species on morphological grounds alone, due to their morphological similarities with other similar species and due to the fact that various strains of one species can demonstrate different morphologies, for example, *Acremonium egyptiacum*, with a pale orange or dull greenish-colored surface, and cylindrical, ellipsoidal, or obovoid conidia in sticky heads or obovoid conidia in dry chains [3]. Currently, the unequivocal identification of the species requires the sequencing of the ITS fungal barcode region, although other targets such as the ribosomal large subunit (LSU), actin, and elongation factor 1-α (EF1-α) have been found phylogenetically informative, and they may thus also serve for the identification of the species by sequencing [8,9].

*Acremonium egyptiacum* has emerged as one of the two main pathogenic *Acremonium* species, the other being *Sarocladium* (previously *Acremonium*) *kiliense* [2,3,6]. In modern taxonomy, *A. egyptiacum* has been synonymized with *Acremonium sclerotigenum*, with both thus occasionally reported as comprising the *Acremonium sclerotigenum*/*egyptiacum* species complex, although the current valid name of the fungus is *A. egyptiacum* [3,8]. *A. egyptiacum* has been isolated from various environmental sources such as soils, seawater/seacoast, plant materials, foods (fishmeal, cucumber, muskmelon, grapefruit juice), and as an endophyte of vines [3]. It has been isolated from both immunocompromised and immunocompetent human subjects, from diseases such as onychomycosis, skin disease, dialysis-related peritonitis, osteomyelitis, and systemic disease, and from clinical sources such as toenails, foot skin, blood, bronchial secretions, peritoneal fluid, tracheal aspirates, sinuses, eye, olecranon bursa, and cerebrospinal fluid [2,3,5,6,10]. In the past scientific literature, it has been occasionally phenotypically misidentified as *Acremonium strictum*, *Acremonium potronii*, or *Acremonium alternatum*, due their shared morphological similarities [2,3].

In Greece, the contribution of acremonium-like species in fungal disease is an unexplored domain. Additionally, there is scant general knowledge of their in vitro susceptibility to antifungal drugs. This is especially a problem concerning *A. egyptiacum*, as it is recognized now as a central pathogenic *Acremonium* species. In that respect, in this study, we accurately identified a collection of 34 acremonium-like clinical strains, isolated over the past 12 years (2010–2021) in Greece, by sequencing the internal transcribed spacer (ITS). We also genotyped the discovered *A. egyptiacum* isolates by determining their ITS barcode types. Furthermore, we determined the susceptibility of our acremonium-like isolates to 10 mainstay antifungal drugs, including the newer topical azoles flutrimazole, lanoconazole, and luliconazole, by employing the CLSI M38 3rd Edition broth microdilution standard method [11].

## 2. Materials and Methods

### 2.1. Strains

We studied a population of 34 *Acremonium* isolates, collected from an equal number of patients in Greece (Athens, Larissa and Thessaloniki) through 2010–2021, which were deposited in the University of Athens/Hellenic Collection of Pathogenic Fungi (UOA/HCPF), Athens, Greece and stored at −80 °C. The vast majority of the isolates originated from toenails (30 isolates), two isolates from inflamed skin, one from a blood culture and one from an inflamed external auditory meatus. The isolates were initially identified as *Acremonium* spp. in a conventional morphological study.

### 2.2. Molecular Identification and Genotyping

Isolates were revived on malt extract agar for 1 week and DNA was extracted as previously described [12]. Briefly, mycelia were transferred into 1.5 mL microcentrifuge tubes containing 500 mL lysis buffer (200 mM Tris-HCl, pH 8, 250 mM NaCl, 25 mM EDTA, 0.5% sodium dodecyl sulfate, all from Sigma, St. Louis, MO, USA), disrupted mechanically with an orbital homogenizer followed by an extraction with a ratio of 25 phenol (Sigma)/24 chloroform (BDH, Poole, UK)/1 isoamyl alcohol (Ferak, Berlin, Germany) and 1 h centrifugation at 4 °C at 13,000× *g*. The supernatant was then removed, and after a pure chloroform extraction, was precipitated with an equal volume of isopropanol (Merk, Darmstadt, Germany) at −20 °C for 15 min and centrifuged at 15,000× *g* for 15 min. The pellet was washed with 70% aqueous solution of ethanol, dried at room temperature, and re-suspended in sterile distilled water. The DNA extracts were stored at 4 °C until processed. Isolates were identified to genus and species level by sequencing of the whole internal transcribed spacer (ITS), comprising the whole of the ITS1 region, the 5.8S rDNA gene, and the whole of the ITS2 region. Forward primers ITS5 or ITS1 and reverse primer ITS4 were used for PCR amplification as previously described [12,13], and the conditions for 35 cycles were 1 min at 95 °C, 1 min at 58 °C, and 1 min 30 s at 72 °C. The final cycle was 1 min at 95 °C, 1 min at 58 °C, and 5 min at 72 °C. The products were directly sequenced at both directions and ITS derived sequences were compared with the GenBank-archived sequences (BLAST; http://www.ncbi.nlm.nih.gov, accessed on 18 September 2024) and were aligned with CLUSTALW [14]. GenBank-derived sequences of type and reference *Acremonium egyptiacum* strains (including the three recognized *Acremonium egyptiacum* genotypes) as per Summerbell et al. [3] were included in the alignment. *Emericellopsis fimetaria*, *Emericellopsis terricola*, and *Emericellopsis tubakii* were employed as the outgroup. The MEGA-X software platform (https://www.megasoftware.net/downloads/dload_win_gui, accessed on 31 July 2024) was used for deriving phylogenetic relations via the neighbor-joining method, the evolutionary distances were computed using the Kimura two-parameter method [15], and the robustness of the trees obtained was evaluated by 1000 bootstrap replications.

### 2.3. Antifungal Susceptibility

Amphotericin B (Sigma, St. Louis, MO, USA), flutrimazole (kindly provided by Galenica S.A., Kifissia, Greece), itraconazole (Johnson and Johnson, Basel, Switzerland), lanoconazole, luliconazole (both kindly provided by Nihon Nohyaku Co., Ltd., Osaka, Japan), posaconazole (Merck, Whitehouse Station, NJ, USA), voriconazole (Pfizer, Sandwich, UK), amorolfine (Manus Aktteva Biopharma LLP, Ahmedabad, India), griseofulvin (Sigma, St. Louis, MO, USA), and terbinafine (Novartis, Basel, Switzerland) minimum inhibitory concentrations (MICs) were recorded by the CLSI M38 Ed3 [11] Reference Method for Broth microdilution Antifungal Susceptibility Testing of Filamentous Fungi. Antifungal drugs were tested in triplicate, according to a modification of CLSI M38 Ed3 guidelines, including testing at higher concentrations from 0.032 to 64 μg/mL. Reference strains *Candida parapsilosis* ATCC^®^ 22019 (Manassas, VA, USA) and *Aspergillus fumigatus* ATCC^®^ MYA-3627 (Manassas, VA, USA), as per CLSI M38 Ed3 guidelines [10], were used at each independent trial.

### 2.4. Statistics

Statistical comparisons amongst drugs for the isolates (n = 18) were made, on log-transformed data, with an analysis of variance (ANOVA) (SPSS v. 16.0; SPSS Inc., Chicago, IL, USA) followed by multiple pair-wise comparisons with Bonferroni corrections.

## 3. Results

### 3.1. Molecular Identification

The molecular identification of the studied population of 34 Acremonium clinical isolates from Greece revealed 18 isolates of *A. egyptiacum* in total (Table 1), according to the NCBI BLAST algorithm (score 99–100%). These comprised about half of the studied population. The majority of these *A. egyptiacum* isolates were isolated from toenails (16 out of 18), and only 1 was isolated from a blood culture and 1 from a plantar skin biopsy. The second most common species was *Sarocladium kiliense* with eight isolates in total, seven deriving from toenails and one from human skin (Table 2). *Acremonium charticola*, *Gliomastix polychroma*, *Proxiovicillium blochii*, *Sarocladium terricola*, *Sarocladium zeae*, and *Stanjemonium dichromosporum* were all represented by one isolate each. This heterogeneous group was all derived from toenails. Two isolates derived from toenails, each with a novel ITS sequence, possibly represent undescribed species, with affinity to Emericellopsis. All derived sequences were deposited to the GenBank under accession numbers OR485204-19, KP132613, GQ376095, KC254084-87, KP132614, PP971123-32, and PP979053 (Table 1 and Table 2).

### 3.2. A. egyptiacum ITS Barcode Types

**Figure 1:**
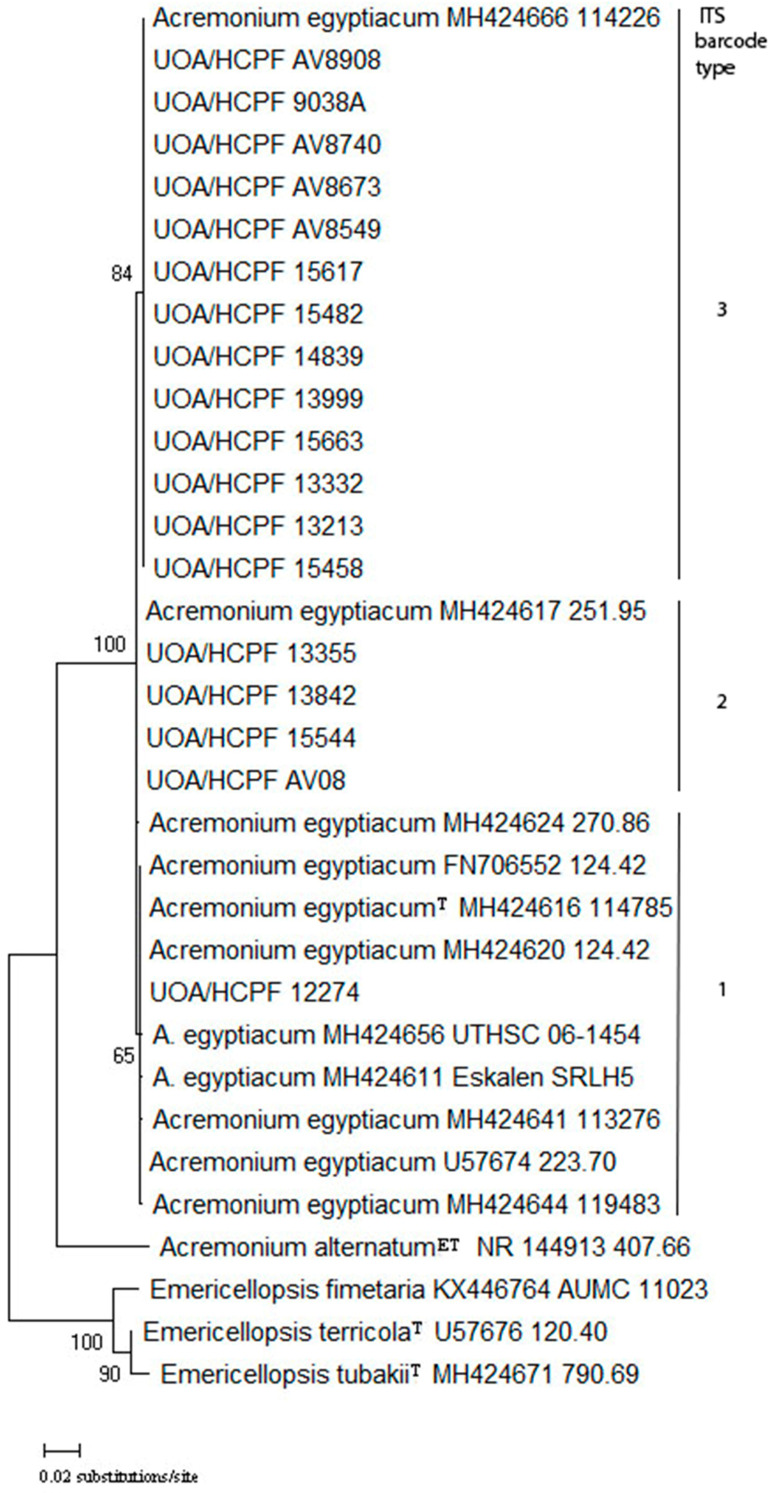
Neighbor-joining tree generated from complete internal transcribed spacer-derived sequences of 18 clinical strains and reference sequences of *Acremonium egyptiacum* strains. Strains belonging to each one of the three ITS barcode types described so far clustered together with the respective reference strains described before. Numbers above branches are bootstrap values.

Among the 18 *A. egyptiacum* isolates revealed by molecular identification, all three ITS barcode types described to date [3] were identified, as seen in Figure 1. The major barcode type was 3 (13 isolates, 72.3%), followed by barcode type 2 (4 isolates, 22.1%), and barcode type 1 (one isolate, 5.6%) (Table 1). Interestingly, no novel barcode type, distinct from the three so far identified, was discovered. The one barcode 1 isolate was isolated from toenails, while from the four isolates comprising barcode 2, three were derived from toenails and 1 from plantar skin biopsy. The majority of barcode 3 isolates were isolated from toenails (n = 12), and only one isolate was isolated from a blood culture (Table 1).

### 3.3. Antifungal Susceptibility

The measured antifungal susceptibility to amphotericin B, flutrimazole, itraconazole, lanoconazole, luliconazole, posaconazole, voriconazole, amorolfine, terbinafine, and griseofulvin is presented in Table 1 and Table 2.

All *A. egyptiacum* isolates showed high amphotericin B MIC (>2 μg/mL) (MIC ranges, geometric mean (GM), and MIC for 50% or 90% of the isolates (MIC50/MIC90) are shown in Table 3). The azole performance was variable, with two groups clearly distinct. The first group comprised flutrimazole, lanoconazole, and luliconazole, with lower MIC values (flutrimazole, GM = 1.71 μg/mL; lanoconazole, GM = 1.41 μg/mL; luliconazole, GM = 1.36 μg/mL). By contrast, the second group, comprising itraconazole, posaconazole, and voriconazole, presented high MIC values (itraconazole, GM = 21.77 μg/mL; posaconazole, GM = 13.7 μg/mL; voriconazole, GM = 17.95 μg/mL). Amorolfine, terbinafine, and griseofulvin MIC (GM, MIC_50_, MIC_90_, all 32 μg/mL) were overall high (MIC > 2 μg/mL) (Table 1 and Table 3). There was no clear correlation of any ITS barcode type with high MICs for all the drugs tested. An analysis of variance performed on absolute values showed that flutrimazole, lanoconazole, and luliconazole were equivalent and notably lower than those of all the other drugs tested against *A. egyptiacum*.

MICs to amphotericin B were overall high (MIC > 2 μg/mL) for all non-*A. egyptiacum* isolates. The azole performance for these isolates was variable with flutrimazole, lanoconazole, and luliconazole presenting the lowest values (flutrimazole, GM, 3.83 μg/mL; lanoconazole, GM, 2.71 μg/mL; luliconazole, GM, 1.83 μg/mL). Itraconazole (GM, 26.91 μg/mL), posaconazole (GM, 21.67 μg/mL), voriconazole (GM, 16.01 μg/mL), amorolfine (GM, 32 μg/mL), terbinafine (GM, 26.91 μg/mL, and griseofulvin (GM, 25.77 μg/mL)) MICs were high. A small number of isolates of various species were found with a comparatively low voriconazole MIC (<4 μg/mL).

## 4. Discussion

This study represents the first survey on clinical *Acremonium* and acremonium-like isolates in Greece, with *A. egyptiacum* and *S. kiliense* being the two dominant species. The clinical dominance of these two species in our isolates is in agreement with their key clinical importance described in a recent review and also with the findings in a recent survey from China [6,16]. There is uncertainty about the ability of other species to cause infections, due to the lack of molecular identification in past case reports describing such species. It is entirely possible though that some of these species will prove to be pathogenic in future studies utilizing the modern taxonomy. In this sense, the finding in our isolates of species reported in past cases, such as *Acremonium* (currently *Proxiovicillium*) *blochii* [17] or *Acremonium roseogriseum* (currently *Giomastix roseogrisea*) [8], or species reported in a recent survey from China [16], such as *Sarocladium terricola* or *Sarocladium zeae*, may show a meaningful consistency.

The only acremonium-like species previously reported in infections from our region was *S. kiliense*, from a fungaemia case [18]. This study is the first report of clinical *A. egyptiacum* from Greece, although one isolate derived from peritoneal fluid was initially identified as A. strictum in 2002 [19] and retrospectively re-identified as *A. egyptiacum* by Summerbell et al. [3]. Our results, in agreement with previous studies [3,16], identify this species as a major nail-related fungal *Acremonium* species, able to function as a pathogen or saprophyte/contaminant. In the past, the fungus has been often reported as *Acremonium strictum* in multiple onychomycosis case reports [2,3]. It has also been reported as *Acremonium potronii* [2,3], a species regarded in the past as the main acremonial cause of onychomycosis but today is regarded as a nomen dubium [3]. In these previous onychomycosis cases, it was mainly clinically related to the white superficial onychomycosis of toenails, a correlation confirmed by recent case reports using the modern nomenclature [20]. *A. egyptiacum* toenail onychomycosis has been reported as the starting point for disseminated disease in a case of aplastic anemia and, consequently, nails should be systematically examined before the initiation of cytotoxic chemotherapy [21].

**Figure 2:**
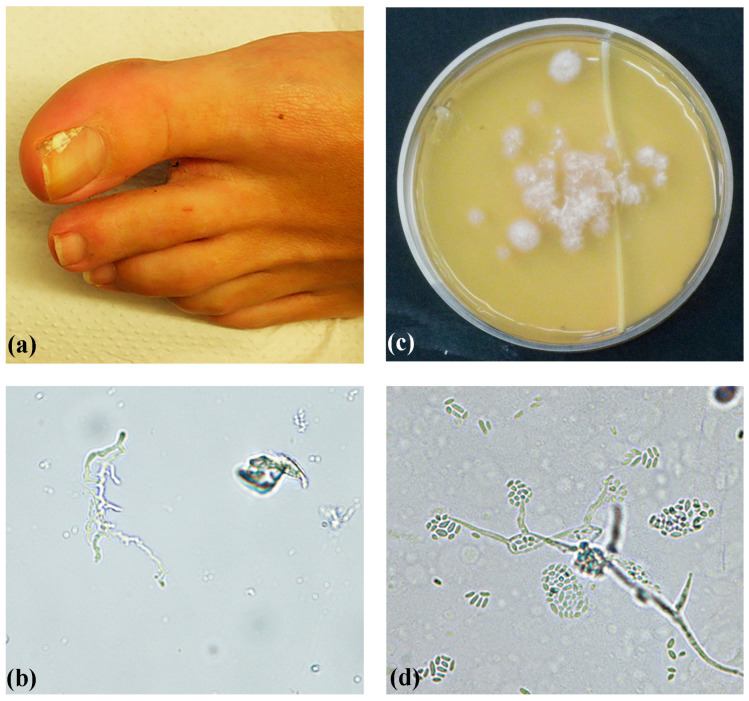
A confirmed (repeated isolation and positive direct microscopy) toenail white superficial onychomycosis case in a 40-year-old woman, caused by a type 3 *Acremonium egyptiacum* isolate. (**a**) White area on the surface of the first toenail. (**b**) Direct microscopy of nail clippings showing a branching hyphal structure (original magnification 400×). (**c**) First isolation directly from nail clippings on Sabouraud dextrose agar, showing multiple *A. egyptiacum* colony forming units, after incubation at 32 °C for 8 days. (**d**) Microscopy showing slimy heads on phialides (original magnification 400×).

In total, three ITS barcode types (genotypes) have been identified thus far in a study examining 32 strains of heterogeneous origin (environmental and clinical) [3]. ITS barcode types 1 and 3 were also found in a recent study from China that identified 14 clinical *A. egyptiacum* strains [16]. Despite the limited *A. egyptiacum* population available in our region in 2010 through to 2021, all three ITS barcode types were found. The majority of our isolates belonged to ITS barcode type 3, in agreement with previous findings that showed a predominance of this type in clinical isolates [3]. An illustrative case of confirmed (repeated isolation and positive direct microscopy) toenail white superficial onychomycosis caused by a type 3 isolate (UOA/HCPF 13213) is presented in Figure 2. In contrast, ITS barcode types 1 and 2 seem to be more common in environmental isolates, although they can also be found in clinical specimens [3]. The one ITS barcode type 1 strain and three out of four type 2 strains were nail isolates. Although type 1 does not grow well or at all at 37 °C and is considered mainly environmental [3], it may grow well in toenails at lower temperatures, from which it is often isolated. Our study is only the third study on ITS barcodes for this fungus [3,16]. Consequently, this topic needs to be studied further due to its clinical (medical and veterinary) importance.

Few studies on the antifungal susceptibility of *A. egyptiacum* have been published until now, probably related to the recent consolidation of its taxonomy and the recent realization of the need for sequencing for its accurate identification [3,9]. Regarding amphotericin B, low in vitro antifungal activity against *A. egyptiacum* isolates has been reported before from China (15 isolates, GM at 12.13) and from France (one isolate, MIC at 12/2 mg/L using the Etest/CLSI method) [16,21]. Similarly, the three systemic azoles tested in this study, itraconazole, posaconazole and voriconazole, showed high MICs, with only a few sporadic isolates demonstrating low MICs to voriconazole and posaconazole. This is in contrast to previously published studies that reported posaconazole and voriconazole as probably the drugs with the best in vitro activity against *A. egyptiacum* [16,21]. These results emphasize the need for establishing CLSI and EUCAST (European Committee on Antimicrobial Susceptibility Testing) clinical break points for this emerging pathogen. The derived high terbinafine MICs constitute another discrepancy with previously published data [16]. The high activity of the flutrimazole, lanoconazole, and luliconazole triad is an encouraging finding, as these drugs are formulations for topical use, and can be used in treating onychomycosis caused by *A. egyptiacum*.

Similarly, the present findings show random high MIC values of all drugs tested for non-*A. egyptiacum* isolates, although the limited sample size of the species in our study does not permit generalizations. There are scant published data on the in vitro susceptibility of non-*A. egyptiacum* species. Regarding *S. kiliense*, in a recent study from China [16], amphotericin B, itraconazole, and posaconazole showed similarly high MICs; in contrast, voriconazole and terbinafine MICs were low. The susceptibility testing against terbinafine was subjected to the limitation of not incorporating terbinafine reference strains. This requires further detailed studies testing a larger population of *Acremonium* isolates. Clearly, the limited information on susceptibilities, the limited data on correlation of in vitro susceptibilities and clinical outcomes, the finding of many isolates with high MICs in our study, and the finding of variable activity in different studies, warrant a more extensive investigation of the antifungal susceptibility of *Acremonium* and acremonium-like clinical isolates, especially to drugs used in the treatment of invasive disease, such as posaconazole and voriconazole.

## 5. Conclusions

In conclusion, *A. egyptiacum* is the dominant medically important Acremonium species in Greece, followed by *Sarocladium kiliense*, *Acremonium charticola*, *Gliomastix polychroma*, *Proxiovicillium blochii*, *Sarocladium terricola*, *Sarocladium zeae*, and *Stanjemonium dichromosporum*. All three *A. egyptiacum* ITS barcode types described to date were identified, and the antifungal susceptibility of different genotypes was homogeneous. The best antifungal activity against *A. egyptiacum* was shown by flutrimazole, lanoconazole, and luliconazole, while amphotericin B, itraconazole, posaconazole, voriconazole, terbinafine, amorolfine, and griseofulvin MICs were overall high. Overall, the high MICs recorded for all systemically administered drugs, and for some topical antifungals against the tested *A. egyptiacum* and other acremonium-like clinical isolates, justify the routine susceptibility testing of clinical isolates, especially from invasive diseases of immunocompromised patients.

## Figures and Tables

**Table 1:** Antifungal susceptibility (CLSI) and ITS barcode types of 18 clinical *Acremonium egyptiacum* isolates.

Strain Identifier (^a^ UOA/HCPF Number)	Origin	Year of Isolation	GenBank Accession (ITS)	ITS Barcode Types ^b^	MIC (μg/mL)									
					AMB ^c^	FLU	ITZ	LAN	LUL	POS	VOR	AML	TER	GRI
9038A	Toenails	2010	GQ376095	3	4	1	8	1	1	8	8	32	8	8
12274	Toenails	2010	OR485204	1	16	4	32	1	1	32	8	32	8	8
12588	Toenails	2010	QR485205	3	8	1	16	1	0.5	4	8	32	16	16
13213	Toenails	2011	OR485206	3	16	32	32	32	32	32	32	32	32	32
13332	Blood culture	2011	QR485207	3	16	1	16	1	1	16	32	32	32	16
13355	Toenails	2011	OR485208	2	16	1	32	1	1	8	16	16	32	32
13842	Plantar skin biopsy	2011	OR485209	2	8	1	16	1	2	16	16	16	32	16
13999	Toenails	2011	QR485210	3	16	1	32	1	2	32	32	32	32	32
14839	Toenails	2014	KP132613	3	16	1	32	2	1	16	32	32	4	16
15458	Toenails	2014	OR485211	3	16	2	32	2	1	32	32	32	8	32
15544	Toenails	2014	OR485212	2	16	1	32	1	1	8	32	32	16	32
15617	Toenails	2015	QR485213	3	16	2	32	2	2	32	32	32	32	32
15663	Toenails	2015	OR485214	3	16	32	32	8	2	32	32	32	32	32
AV08	Toenails	2012	OR485215	2	16	1	32	2	0.5	32	32	32	32	32
AV8549	Toenails	2015	QR485116	3	8	0.25	2	0.25	1	0.25	0.5	32	32	8
AV8673	Toenails	2015	OR485217	3	8	2	32	1	1	8	32	8	32	32
AV8740	Toenails	2020	OR485218	3	8	1	32	1	1	16	16	32	32	32
AV8908	Toenails	2021	QR485219	3	16	2	16	0.5	2	32	32	32	32	32

^a^ University of Athens/Hellenic Collection of Pathogenic Fungi. ^b^ As defined in Summerbell et al. [3]. ^c^ AMB, amphotericin B; FLU, flutrimazole; ITZ, itraconazole; LAN, lanoconazole; LUL, luliconazole; POS, posaconazole; VOR, voriconazole; AML, amorolfine; TER, terbinafine; GRI, griseofulvin.

**Table 2:** Identification and antifungal susceptibility (CLSI) of 16 clinical non-*Acremonium egyptiacum* isolates.

Strain Identifier (^a^ UOA/HCPF Number)	Origin	Year of Isolation	GenBank Accession (ITS)	Identification	MIC (μg/mL)									
					AMB ^b^	FLU	ITZ	LAN	LUL	POS	VOR	AML	TER	GRI
6229	Toenails	2011	PP971132	*Gliomastix polychroma*	16	32	32	32	1	32	32	32	32	32
11985	Toenails	2011	KC254086	*Sarocladium kiliense*	16	4	32	2	1	32	2	32	32	8
11624	Toenails	2011	KC254085	*Sarocladium kiliense*	16	32	32	1	2	32	32	32	32	32
11447	Toenails	2011	KC254084	*Sarocladium kiliense*	16	2	32	32	32	32	32	32	32	32
12768A	Toenails	2011	KC254087	*Sarocladium kiliense*	16	1	32	1	1	8	16	32	32	32
13246	Toenails	2011	PP971127	*Proxiovicillium blochii*	16	1	32	1	2	32	4	32	32	32
13447	External otitis	2011	PP971123	*Sarocladium kiliense*	16	32	32	32	32	32	32	32	32	32
13520	Human skin	2011	PP971124	*Sarocladium kiliense*	16	32	32	32	32	32	32	32	32	32
15442	Toenails	2014	PP971131	*Emericellopsis* sp.	16	4	16	2	1	32	16	32	8	32
15471	Toenails	2014	PP971125	*Sarocladium kiliense*	8	2	16	1	1	8	32	32	16	32
15482	Toenails	2014	PP971126	*Sarocladium terricola*	8	1	16	1	0.5	8	4	32	16	16
15618	Toenails	2014	PP979053	*Sarocladium kiliense*	16	32	32	1	1	32	32	32	32	32
15589	Toenails	2014	PP971129	*Stanjemonium dichromosporum*	8	1	32	1	2	16	32	32	32	16
AB1112	Toenails	2021	KP132614	*Sarocladium zeae*	16	1	32	2	0.5	32	32	32	32	32
AV1768	Toenails	2012	PP971130	*Emericellopsis* sp.	16	1	32	1	0.25	32	8	32	32	32
AV7579	Toenails	2014	PP971128	*Acremonium charticola*	8	1	16	1	1	8	8	32	32	16

^a^ University of Athens/Hellenic Collection of Pathogenic Fungi. ^b^ AMB, amphotericin B; FLU, flutrimazole; ITZ, itraconazole; LAN, lanoconazole; LUL, luliconazole; POS, posaconazole; VOR, voriconazole; AML, amorolfine; TER, terbinafine; GRI, griseofulvin.

**Table 3:** Minimum inhibitory concentration (MIC) ranges, geometric means (GM), and distributions for 50% (MIC_50_) and 90% (MIC_90_) of 18 *Acremonium egyptiacum* isolates, determined by CLSI M38 Ed3 standard microdilution method for filamentous fungi. All units of measurement are expressed in μg/mL.

	AMB ^a^	FLU	ITZ	LAN	LUL	POS	VOR	AML	TER	GRI
**MIC** **range**	4–16	0.25–32	2–32	0.25–32	0.5–32	0.25–32	0.5–32	8–32	4–32	8–32
**GM**	12.21	1.71	21.77	1.41	1.36	13.7	17.95	27.43	20.95	21.77
**MIC_50_/MIC_90_**	16/16	1/4	32/32	1/2	1/2	8/32	32/32	32/32	32/32	32/32

^a^ AMB, amphotericin B; FLU, flutrimazole; ITZ, itraconazole; LAN, lanoconazole; ITZ, luliconazole; POS, posaconazole; VOR, voriconazole; AML, amorolfine; TER, terbinafine; GRI, griseofulvin.

## Data Availability

All derived sequences were deposited to GenBank under accession numbers OR485204-19, KP132613, GQ376095, KC254084-87, KP132614, PP971123-32, and PP979053.
